# Anion gap, anion gap corrected for albumin, base deficit and unmeasured anions in critically ill patients: implications on the assessment of metabolic acidosis and the diagnosis of hyperlactatemia

**DOI:** 10.1186/1471-227X-8-18

**Published:** 2008-12-16

**Authors:** Lakhmir S Chawla, Shirley Shih, Danielle Davison, Christopher Junker, Michael G Seneff

**Affiliations:** 1 Department of Critical Care Medicine and Anesthesiology, George Washington University Medical Center, Washington DC, USA; 2 Division of Renal Diseases and Hypertension, Department of Medicine, George Washington University Medical Center, Washington DC, USA

## Abstract

**Background:**

Base deficit (BD), anion gap (AG), and albumin corrected anion gap (ACAG) are used by clinicians to assess the presence or absence of hyperlactatemia (HL). We set out to determine if these tools can diagnose the presence of HL using cotemporaneous samples.

**Methods:**

We conducted a chart review of ICU patients who had cotemporaneous arterial blood gas, serum chemistry, serum albumin (Alb) and lactate(Lac) levels measured from the same sample. We assessed the capacity of AG, BD, and ACAG to diagnose HL and severe hyperlactatemia (SHL). HL was defined as Lac > 2.5 mmol/L. SHL was defined as a Lac of > 4.0 mmol/L.

**Results:**

From 143 patients we identified 497 series of lab values that met our study criteria. Mean age was 62.2 ± 15.7 years. Mean Lac was 2.11 ± 2.6 mmol/L, mean AG was 9.0 ± 5.1, mean ACAG was 14.1 ± 3.8, mean BD was 1.50 ± 5.4. The area under the curve for the ROC for BD, AG, and ACAG to diagnose HL were 0.79, 0.70, and 0.72, respectively.

**Conclusion:**

AG and BD failed to reliably detect the presence of clinically significant hyperlactatemia. Under idealized conditions, ACAG has the capacity to rule out the presence of hyperlactatemia. Lac levels should be obtained routinely in all patients admitted to the ICU in whom the possibility of shock/hypoperfusion is being considered. If an AG assessment is required in the ICU, it must be corrected for albumin for there to be sufficient diagnostic utility.

## Background

The use of anion gap assessment to interpret and diagnose the etiology of metabolic acidosis was originally described by Emmet and Narins in 1977.[1] Lactic acid, a "gap" acid, is one cause of elevated anion gap metabolic acidosis, and an elevated serum lactate level has emerged as an important tool to screen for patients in shock. Elevated serum lactate can be caused by inadequate perfusion, but may also be a product of inflammation, cytopathic hypoxia, and increased rates of glycolysis. [2-4] In critically ill patients, an elevated lactate level is indicative of increased severity of illness and subsequent serum lactate clearance predicts an improved outcome.[5,6] Rivers et al, utilized hypotension and elevated serum lactate levels to identify patients in shock and demonstrated that emergency department patients with presumed sepsis and a serum lactate level of ≥ 4.0 mmol/L and/or frank hypotension are at a significant risk of death (38–59% mortality).[7] Despite this study and multiple other investigations that document the value of measuring serum lactate concentrations, the measurement of serum lactate is still not routine. In fact, in some institutions, serum lactate remains a "send out" test (unpublished data, Table 1). We believe that one reason the measurement of serum lactate is not part of a standard admission battery of laboratory tests is that clinicians assume other commonly measured and calculated lab values, such as anion gap (AG) and base deficit (BD), accurately identify the presence or absence of hyperlactatemia. Despite previous studies showing that neither base deficit nor anion gap are effective at discriminating between the presence or absence of hyperlactatemia, [8-12] there persists the commonly held belief that a normal anion gap or the absence of base deficit rules out the presence of hyperlactatemia.

**Table 1:** Availability of serum lactate in Washington DC metro area hospitals

**Hospital**	**# of ICU Beds**	**Lac run on-site**	**Lac run automatically with ABG?**	**Serum lactate must be ordered separately?**
George Washington University Hospital	45	Yes	Yes	Separate or comes with ABG
Georgetown University Hospital	25	Yes	Yes	Separate or comes with ABG
Washington Hospital Center	60	Yes	No	Yes
Holy Cross Hospital	42	Yes	No	Yes
Sibley Hospital	14	Yes	No	Yes
Suburban Hospital	44	Yes	No	Yes
Providence Hospital	17	Yes	No	Yes
Specialty Hospital	22	No	No	Yes
Fairfax Hospital	60	Yes	No	Yes
Alexandria Hospital	30	Yes	Yes	Separate or comes with ABG
Washington Adventist Hospital	36	Yes	No	Yes

One possible reason for this discrepancy is that hypoalbuminemia, a common finding in critically ill patients, can cause a decrease in the **"**normal" measured anion gap and thereby mask the presence of an elevated anion gap.[13] Therefore, some investigators have suggested that anion gap corrected for albumin (ACAG) is a more appropriate screening tool for the diagnosis of metabolic acidosis in the ICU.[14] We recently published a study wherein we describe the limited utility of anion gap, anion gap corrected for albumin, and base deficit to diagnose the presence of hyperlactatemia in critically ill patients.[15] In that study, we based our retrospective analysis on laboratory results that were obtained on admission to the intensive care unit (ICU). The major limitation of that study was the fact that we could not be certain if the measured values were drawn contemporaneously. We set out to verify the results of this and other previous studies, using cotemporaneous arterial samples in a larger and more diverse population of critically ill patients.

## Methods

This study was conducted from September 2005 to August 2006 in the George Washington University Hospital ICU. This ICU is a closed, 48 bed combined medical-surgical unit that admits all critically ill adults, except those with major thermal injuries. A waiver of informed consent and HIPPA was obtained from the Institutional Review Board (IRB) because the study involved prospective chart review only. We obtained a HIPAA waiver from the George Washington University Committee on Human Research and the privacy officer of the hospital.

### Patients

We reviewed the records of all medical-surgical ICU admissions over a 12-month time span. Demographic, admission diagnoses, clinical, and biochemical data were collected from the chart for all patients entered into the cohort. We enrolled patients who had arterial lines in place as part of their ICU care and who also had cotemporaneous arterial blood gas, serum chemistry, serum albumin and a serum lactate level measured from the same sample available for review. Patients with a serum creatinine > 6.0 mg/dl, a diagnosis of ketoacidosis, or with a recent history or syndrome consistent with a toxic ingestion (e.g. ethanol, ethylene glycol, methanol, salicylate**s**, toluene, citrate, iron, or isoniazid), and those treated with renal replacement therapy were excluded.

### Definitions and Analysis

For each patient, standard base deficit, anion gap, and anion gap corrected for serum albumin were calculated. Standard base deficit (BD) was determined using the modified Van Slyke equation.[16] Anion gap (AG) was calculated using the formula [Na] - ([Cl] + [HCO3]). Albumin corrected anion gap (ACAG) was calculated using the Figge equation: ({4.4 - [observed serum albumin (g/dL)] × 2.5} + AG).[13] Hyperlactatemia was defined as a serum lactate concentration > 2.5 mmol/L (1.0 mmol/L above our lab's upper limit of normal), and *severe *hyperlacatatemia was defined as a serum lactate > 4.0 mmol/L. Anion gap corrected for albumin and serum lactate (ALCAG) was calculated with the following equation: ({4.4 - [observed serum albumin (g/dL)] × 0.25} + AG) - [serum lactate (mmol/L)]. Patients with a serum creatinine less than or equal to 2.0 mg/dl were also analyzed separately.

### Statistics

Proportions of patients with certain characteristics were compared using the chi-square test. We assessed the distribution of variables. AG, BD, and ACAG were compared using Pearson correlations. Receiver operating characteristic (ROC) curves were determined for AG, BD, and ACAG to detect the presence of hyperlactatemia. Unless otherwise specified, all means are reported as ± S.D. All statistics were performed with SPSS 11.0 (SPSS, Chicago, IL.). The cohort was analyzed with all of the samples from each of the patients, and the cohort was analyzed with only one sample from each patient in order to ascertain if the samples per subject skewed the results.

## Results

We reviewed 1300 consecutive admissions to the ICU from September 2005 to August 2006. One hundred and forty three patients met our inclusion/exclusion criteria. From those patients we identified 497 series of lab values that had an ABG, serum chemistry, and a serum lactate measured from the same arterial sample available for review. Of the 497 subjects, 311 also had a cotemporaneous serum albumin available. The mean age was 62.2 ± 15.7 years and 41.3% of the patients were female. Within the cohort, 51.0% of the patients were African American, 42.7% of the patients European American, 4.9% of the patients Hispanic, and 0.7% of the patients Asian American. Among the 497 sets of laboratory results, hyperlactatemia was present in 16.3% of the patients based on the initial lab values. The serum lactate range was 0.5 to 17.0 mmol/L and the mean serum lactate was 2.11 ± 2.6 mmol/L. The mean serum albumin was 2.5 ± 0.80 g/dl, mean anion gap was 9.0 ± 5.1 meq/L, mean ACAG was 14.1 ± 3.8 meq/L, mean BD was 1.50 ± 5.35, and mean ALCAG was 12.6 ± 3.60 meq/L, Table 2. Sensitivity, specificity, and ROC area under the curve for AG, BD, and ACAG for varying serum lactate thresholds are presented in Tables 3 and 4. Similar analyses where conducted in the patients with a serum creatinine of ≤ 2.0 mg/dl (Table 6). These results did not significantly differ from those of the entire cohort. In addition, the analysis of using each patient to contribute only one sample to the cohort were not significantly different from the results presented (data not shown).

**Table 2:** Demographics and patient characteristics

Age, years	62.2 (15.7)
Gender (% male)	59.7%
Ethnicity	
European American	42.7%
African American	51.0%
Hispanic	4.9%
Asian American	0.7%
Surgical Patients	56.2%
Serum Albumin, g/dL	2.5 (0.86)
Serum lactate, mmol/L	2.11 (2.6)
Base Deficit	1.50 (5.4)
Anion Gap	9.0 (5.1)
Albumin Corrected Anion Gap	14.1 (3.8)
APACHE II Score	12.8 (7.7)
	
Mean (s.d.)	

**Table 3:** Sensitivity, specificity and ROC area under the curve for AG, ACAG & BD.

Variable	ROC AUC	CIs		
		
Anion Gap	0.70	0.64 – 0.77		
ACAG	0.72	0.62 – 0.82		
Base Deficit	0.79	0.73 – 0.85		
				
Variable	Threshold	Sensitivity	Specificity	NPV
Anion Gap	10	63.0%	65.4%	90.0%
	12	51.9%	80.0%	90.0%
	14	39.5%	88.7%	88.2%
	16	27.2%	94.0%	87.0%
				
ACAG	10	94.4%	15.5%	99.4%
	12	88.9%	29.2%	95.3%
	14	75.0%	53.5%	94.2%
	16	55.6%	74.9%	92.8%
				
Base Deficit	2	81.5%	66.8%	94.9%
	4	63.0%	80.4%	91.8%
	6	50.6%	88.1%	90.2%
	8	40.7%	91.6%	88.8%

Hyperlactatemia defined as serum lactate > 2.5 mmol/L

ROC AUC = Receiver operator characteristics area under the curve, AG = anion gap, ACAG = anion gap corrected for albumin, BD = Base Deficit, CI = 95% confidence intervals, NPV = negative predictive value

**Table 4:** Sensitivity, specificity and ROC area under the curve for AG, ACAG & BD.

Variable	ROC AUC	CIs		
		
Anion Gap	0.87	0.82 – 0.93		
ACAG	0.95	0.91–0.99		
Base Deficit	0.78	0.71 – 0.86		
				
Variable	Threshold	Sensitivity	Specificity	NPV
Anion Gap	10	88.9%	65.7%	98.3%
	12	82.2%	80.4%	97.8%
	14	66.7%	89.2%	96.4%
	16	48.9%	94.5%	94.9%
				
ACAG	10	100%	15.1%	100%
	12	100%	29.8%	100%
	14	100%	51.5%	100%
	16	92.9%	74.4%	99.5%
				
Base Deficit	2	84.4%	63.3%	97.6%
	4	64.4%	77.1%	95.6%
	6	51.1%	84.5%	94.5%
	8	42.2%	89.5%	93.9%

Hyperlactatemia defined as Lactate > 4.0 mmol/L

ROC AUC = Receiver operator characteristics area under the curve, AG = anion gap, ACAG = anion gap corrected for albumin, BD = Base Deficit, CI = 95% confidence intervals, NPV = Negative Predictive Value

**Table 5:** Summary of previous studies

**Anion Gap Studies**				
				
Study	N	Sensitivity	Specificity	ROC
Iberti et al[8]	56	21%	*Not Reported*	*Not Reported*
Levraut et al[10]	498	44%	91%	0.79
Moviat et al[18]	50	45%	16%	*Not Reported*
Dinh et al[19]	356	39%	89%	0.76
Chawla et al[15]	285	15%	94%	0.55
				
**Anion Gap Corrected for Albumin Studies**				
				
Study	N	Sensitivity	Specificity	ROC

Moviat et al[18]	50	100%	11%	*Not Reported*
Dinh et al[19]	356	75%	59%	0.75
Chawla et al[15]	285	32%	80%	0.57
				
**Base Deficit Studies**				
				
Study	N	Results		

Mikulaschek et al[11]	52	No correlation between lactate and base deficit		
Waters et al [11,24]	12	Base deficit not useful, instead misleading		
Chawla et al[15]	285	Base Deficit not useful, ROC AUC = 0.64		

ROC = Receiver operator characteristics area under the curve, AG = anion gap, ACAG = anion gap corrected for albumin, BD = Base Deficit, sensitivity and specificity for AG and ACAG are reported at threshold of 12

**Table 6:** Subset of patients with creatinine > 2.0 mg/dl excluded

ROC Area Under the Curve for AG, ACAG & BD *Hyperlactatemia defined as serum lactate > 2.5 mmol/L*		
		
Variable	ROC AUC	CIs
Anion Gap	0.68	0.59 – 0.77
ACAG	0.71	0.59 – 0.84
Base Deficit	0.77	0.69 – 0.85
		
ROC Area Under the Curve for AG, ACAG & BD *Hyperlactatemia defined as serum lactate > 4.0 mmol/L*		
		
Variable	ROC AUC	CIs

Anion Gap	0.90	0.83 – 0.97
ACAG	0.96	0.91–0.99
Base Deficit	0.75	0.62 – 0.88

## Discussion

In this study, we showed that base deficit (BD) and anion gap (AG) are poor tests to diagnose the presence of hyperlactatemia (serum lactate > 2.5 mmol/L). AG has a clinical threshold of 10–12 meq/L. At these values, AG performs quite poorly in predicting the presence of hyperlactatemia with a sensitivity of 63% and a specificity of 80.0% (Table 3). When the threshold of serum lactate is elevated to 4.0 mmol/L, the sensitivity improves to 88.9% and the specificity to 80.4%, but these levels remain unsatisfactory to be clinically reliable. Unlike AG and BD, ACAG performs much better for diagnosing the *presence *of hyperlactatemia. The diagnostic performance of ACAG improves considerably with the sensitivity increasing from 63% to 94.4% as compared to the AG (Table 3); however, this improvement in sensitivity comes at the cost of a low specificity (29.2%). When the threshold for serum lactate increases to 4.0 mmol/L, the sensitivity of ACAG improves to 100% (Table 4), but the specificity remains poor (29.8%). As a practical matter, the negative predictive value for ACAG and BD was satisfactory (> 88%), and may have utility as a tool to rule out the presence of hyperlactatemia.

In order to assess the performance of a test across its diagnostic range, ROC curves are useful. Typically, a test with a high ROC area under the curve signifies a good diagnostic test, and a point on the curve with a high sensitivity and specificity can be selected for diagnostic purposes. In the case the anion gap (AG or ACAG) the cut-off point has been determined by clinical practice (10–12 meq/L). At this preset threshold, AG does not perform well enough to be clinically reliable (Table 3). However, ACAG can be used for the purpose of ruling out the presence for hyperlactatemia and severe hyperlactatemia (Table 4). Yet, it is important to recognize that if serum albumin is not measured contemporaneously with the serum electrolytes, this relationship does not hold as evidenced by our previous study.[15]

In contrast to our previous study when we assessed ICU admission lab data.[15], BD, AG, and ACAG perform significantly better when the serum lactate, blood gas, and serum electrolytes are drawn from the same sample. Despite this relative improvement, neither AG nor BD possess adequate diagnostic capacity for routine clinical use to rule in or rule out hyperlactatemia, a finding consistent with previous investigations. Iberti et al showed in a cohort of critically ill patients that only 21% of patients with a serum lactate level between 2.5 mmol/L and 4.9 mmol/L had an elevated anion gap, consistent with other studies.[8,10-12] Other studies have shown that as the serum lactate rises to 4.0–5.0 mmol/L, an elevated anion gap and base deficit become more specific at detecting *severe *hyperlactatemia.[10,17] The performance of ACAG to diagnose the presence of hyperlactatemia has been assessed in two limited previous studies. Moviat et al showed in small series of samples of critically patients with metabolic acidosis that ACAG had improved sensitivity but worse specificity for detecting the presence of hyperlactatemia. We verify these findings of Moviat[18] et al in a larger (497 samples compared to 50) more diverse population of critically ill patients. Additionally, we tested the sensitivity and specificity in varying thresholds of serum lactate. Dinh[19] et al conducted a retrospective study in a large cohort of hospitalized patients. In that study, ACAG was no better than AG in predicting the presence of hyperlactatemia, and both were shown to be poor diagnostic tools for the diagnosis of hyperlactatemia. In contrast to their study, our study assessed arterial samples as opposed to peripheral venous samples; arterial samples are fully 'mixed' and less apt to regional error (e.g. tourniquet effects during phlebotomy, differences in limb flow and oxygen consumption etc.). We were also able to assess the performance of base deficit. A review of previous studies' assessment of BD, AG, and ACAG for the diagnosis of hyperlactatemia is provided in Table 5.

The implications of these data are noteworthy. Because elevated serum lactate levels identify patients who are at high risk of death and may identify patients in shock before they become hypotensive (a condition called cryptic shock), early recognition and treatment of hyperlactatemia is critical, and likely improves mortality.[7] In order to institute appropriate therapy as timely as possible, screening tests for shock should offer as early a warning as possible, well before the serum lactate rises to 4.0–5.0 mmol/L. For these reasons, the routine use of AG, BD, and ACAG as screening tests to determine the presence or absence of hyperlactatemia, in our opinion, is unacceptable and potentially harmful. While it is true that the AG and BD detect the presence of hyperlactatemia more effectively as the threshold value for lactate is raised (serum lactate > 4.0 mmol/L), waiting to diagnose hyperlactatemia by allowing the level to rise may delay appropriate intervention. An ACAG < 10 meq/L appears to effectively rule out the presence of hyperlactatemia, but the serum albumin and serum electrolytes must be cotemporaneous and from the same sample in order for that relationship to be valid. Given that accurate and rapid serum lactate concentration measurement is now widely available to all major hospitals (central labs and/or point of service testing), serum lactate concentrations should be routinely measured upon admission to the ICU, for many patients in the emergency department, and in our opinion should be considered an index laboratory measure. Serum lactate remains an assay that must be requested separately in most ICUs and emergency departments; therefore, a clinician must actively ask for this test (Table 1). Further, the use of anion gap and base deficit to diagnose the presence or absence of hyperlactatemia is still commonly taught to medical students and physicians in training. As clinicians and teachers, we need to correct this misperception in order to identify patients with hyperlactatemia promptly.

**Figure 1:**
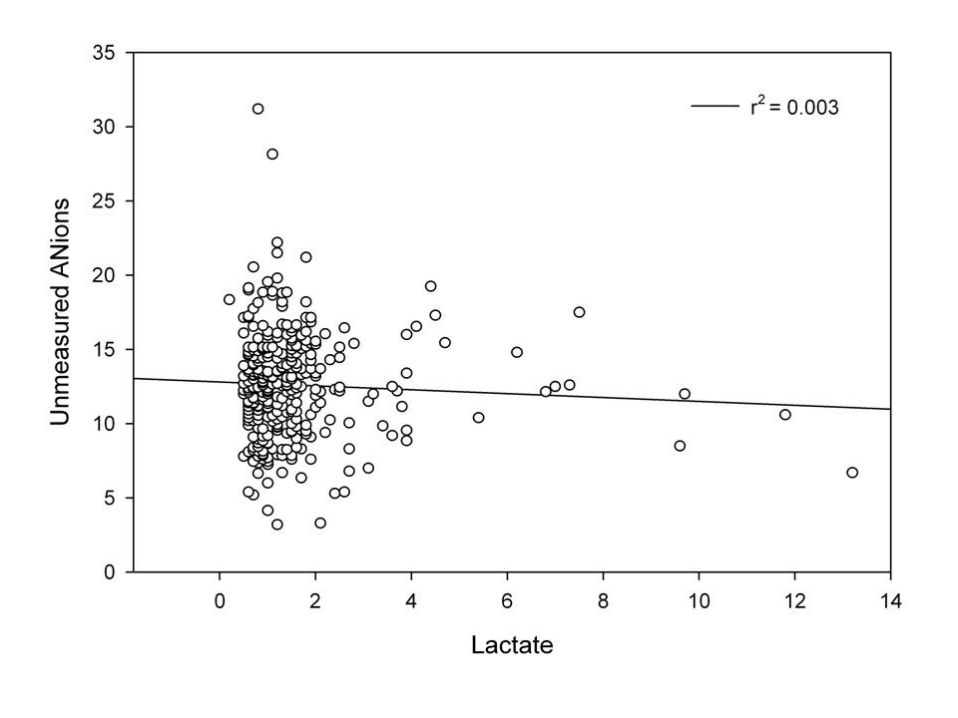
**Albumin lactate corrected anion gap (ALCAG) v. serum lactate**.

**Figure 2:**
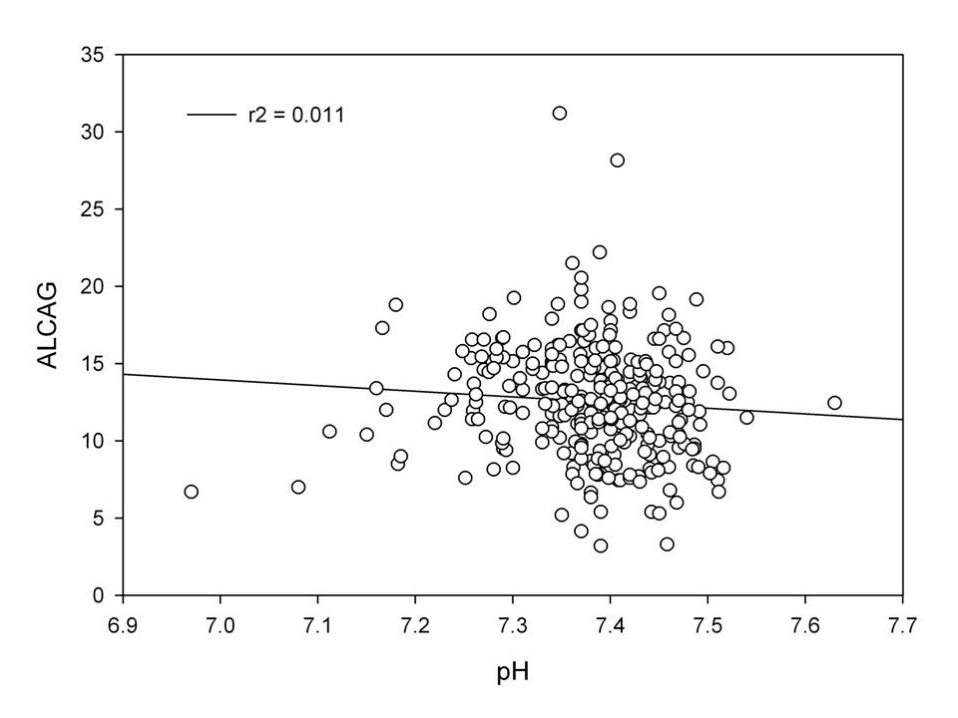
**Albumin lactate corrected anion gap (ALCAG) v. pH**.

In this study, the shortcomings of using the AG to assess metabolic acidosis were exposed. As expected, the sensitivity of anion gap improves when the anion gap is corrected for albumin (ACAG). However, the specificity of the ACAG remained low. The reason for this is illustrated in Figure 1 and Figure 2. In these plots, ALCAG (albumin and lactate corrected anion gap) is plotted against the serum lactate (Figure 1) and pH (Figure 2). Throughout the range of serum lactate and pH, the quantity of unmeasured anions is considerable. The mean ALCAG for the entire cohort was 12.6 ± 3.61. Given that patients with toxic, ingestions, uremia, and those with ketoacidosis were excluded and that lactate and serum albumin are accounted for in the ALCAG equation, the amount of unmeasured anions in this cohort of critically ill patients is elevated. In order to better quantify these anions, the Fencl-Stewart methodology for acid-base assessment would be preferable.[20] In this approach, the unmeasured anions can be assessed because the strong ion difference (apparent and effective) is directly measured. This methodology involves the cotemporaneous measurement of the serum sodium, potassium, magnesium, chloride, lactate, pH, phosphorus, pCO_2_, and serum albumin.[21] In this cohort, the magnesium, phosphorus, and calcium were not consistently available in order to measure the true quantity of the unmeasured anions (strong ion gap). Because we do not have all the requisite information to calculate the strong ion gap (SIG), we cannot be certain that the ALCAG is representative of the SIG. In one small study, the SIG and the ALCAG were highly correlated (r^2 ^= 0.934, p < 0.0001).[18] We hypothesize that the ALCAG may be an easy bedside measurement that may approximate the SIG in patients who are critically ill. Further research of this relationship in large diverse populations is warranted. The etiology of the unmeasured anions commonly found in critically ill patients has been described. In patients with lactic acidosis and in patients with 'unexplained anion gap acidosis' and normal serum lactates, plasma concentrations of acids associated with the Krebs cycle are significantly elevated.[22] The unmeasured identified anions are: citrate, isocitrate, α-ketoglutarate, succinate, maleate, and d-lactate. Because these acids are often tri- and di- basic, smaller concentrations can have larger effects on the anion gap. Unmeasured anions as quantified by the Fencl-Stewart methodology predict outcomes in critically ill patients better than serum lactate.[23] The etiology for why these anions are increased in critically ill patients is unknown, but mitochondrial dysfunction and cellular cytopathic hypoxia as well as disordered glycolytic effects have been proposed.[22] We speculate that the quantity of these unmeasured anions in relationship to the serum lactate (unmeasured anions to lactate ratio) may provide a means for assessing the etiology of lactic acidosis and/or as a predictor of mortality. Further research is warranted.

### Limitations

Our study was conducted in 143 patients with over 497 cotemporaneous samples. Ideally we would have been able to perform this study in a larger population in order to maximize our power. In addition, physical chemistry assessments for unmeasured anions could not be conducted in this cohort. Validation of these data in future cohorts of patients will need to be conducted.

## Conclusion

AG, ACAG, and BD failed to detect the presence of clinically significant hyperlactatemia. The assessment of AG in critically ill patients is highly limited given the prevalence of hypoalbuminemia. If an assessment of the AG is needed, it should be done in concert with serum albumin and serum lactate measurements (ACAG and ALCAG). We believe that serum lactate levels should be routinely obtained in all patients admitted to the ICU in whom the possibility of shock/hypoperfusion is being considered. Unmeasured anions exclusive of serum lactate and serum albumin are frequently present in significant quantities in patients who are critically ill.

## Competing interests

The authors declare that they have no competing interests.

## Authors' contributions

SS participated in the conduct, design, and data acquisition of the study. DD conducted the surveys, and helped draft the manuscript. CJ participated in its design and coordination of the study. MS participated in the design of the study and helped to draft the manuscript. LC conceived of the study, participated in its design and coordination, performed the statistical analysis, and helped to draft the manuscript. All authors read and approved the final manuscript.

## About the authors

LC is a nephrologist and intensivist.

SS is a general surgeon.

DD is an intensivist.

CJ is an anesthesiologist and an intensivist.

MS is the Director of the ICU at George Washington University Hospital.

## Pre-publication history

The pre-publication history for this paper can be accessed here:


